# Visual feedback and motor memory contributions to sustained motor control deficits in autism spectrum disorder across childhood and into adulthood

**DOI:** 10.21203/rs.3.rs-4831158/v1

**Published:** 2024-09-04

**Authors:** Robin L. Shafer, James Bartolotti, Abigail Driggers, Erin Bojanek, Zheng Wang, Matthew W. Mosconi

**Affiliations:** University of Kansas; University of Kansas Medical Center; University of Kansas; University of Rochester School of Medicine and Dentistry; University of Florida; University of Kansas

**Keywords:** Visuomotor, visual feedback, motor memory, autism spectrum disorders, sensorimotor, sensory integration, fine motor control, entropy, grip force

## Abstract

**Background:**

Autistic individuals show deficits in sustained fine motor control which are associated with an over-reliance on visual feedback. Motor memory deficits also have been reported during sustained fine motor control in autism spectrum disorders (ASD). The development of motor memory and visuomotor feedback processes contributing to sustained motor control issues in ASD are not known. The present study aimed to characterize age-related changes in visual feedback and motor memory processes contributing to sustained fine motor control issues in ASD.

**Methods:**

Fifty-four autistic participants and 31 neurotypical (NT) controls ages 10–25 years completed visually guided and memory guided sustained precision gripping tests by pressing on force sensors with their dominant hand index finger and thumb. For visually guided trials, participants viewed a stationary target bar and a force bar that moved upwards with increased force for 15s. During memory guided trials, the force bar was visible for 3s, after which participants attempted to maintain their force output without visual feedback for another 12s. To assess visual feedback processing, force accuracy, variability (standard deviation), and regularity (sample entropy) were examined. To assess motor memory, force decay latency, slope, and magnitude were examined during epochs without visual feedback.

**Results:**

Relative to NT controls, autistic individuals showed a greater magnitude and steeper slope of force decay during memory guided trials. Across conditions, the ASD group showed reduced force accuracy (β = .41, R^2^ = 0.043, t_79.3_=2.36, p = 0.021) and greater force variability (β=−2.16, R^2^ = .143, t_77.1_=−4.04, p = 0.0001) and regularity (β=−.52, R^2^ = .021, t_77.4_=−2.21, p = 0.030) relative to controls at younger ages, but these differences normalized by adolescence (age × group interactions). Lower force accuracy and greater force variability during visually guided trials and steeper decay slope during memory guided trials were associated with overall autism severity.

**Conclusions:**

Our findings that autistic individuals show a greater rate and magnitude of force decay than NT individuals following the removal of visual feedback indicate that motor memory deficits contribute to fine motor control issues in ASD. Findings that sensorimotor differences in ASD were specific to younger ages suggest delayed development across multiple motor control processes.

## Introduction

Sensorimotor impairments are highly prevalent in autism spectrum disorder (ASD) (1). They are associated with the severity of core social, communication, and repetitive behaviors, as well as cognitive outcomes (2–6), and they are some of the earliest signs of atypical development in children who later receive a diagnosis of ASD (2, 7). Sensorimotor differences in autistic persons have been observed across multiple behaviors and effector systems (8–13), and they involve multiple motor control processes including motor planning (10, 13), online motor control (10, 13), and motor learning (4, 14, 15). Additionally, structural and functional differences in cerebellar-cortical brain networks involved in sensorimotor control repeatedly have been observed in ASD (15–21). These findings highlight an important role of sensorimotor differences in ASD and support the need to identify motor control and neurodevelopmental mechanisms of sensorimotor impairments in autistic individuals.

Deficits of sustained, or online sensorimotor control, including the ability to reactively adjust motor output in response to sensory feedback, have been repeatedly shown in ASD. During tests of visually guided precision gripping, our lab has demonstrated that autistic individuals have increased variability and regularity of sustained grip force relative to neurotypical controls, suggesting that the ability to use sensory feedback to make precise and accurate corrective adjustments to ongoing movements is impaired (10, 17, 22, 23), though a separate study of 22 autistic individuals suggested that elevations in force variability were specific to, or at least more severe, during tasks with a dynamic (moving) target rather than a static (fixed) target (24). These deficits are exacerbated when the spatial resolution of visual feedback is enhanced or degraded, indicating that autistic persons are over-reliant on visual feedback to correct error in grip force (10, 17, 23). Autistic individuals also show reduced effects of somatosensory feedback manipulations (tendon vibration) on grip force control relative to controls, regardless of the resolution of visual feedback, suggesting that autistic persons have reduced reliance on secondary sensory inputs during sensory feedback guided motor behaviors (22).

Sustained motor control also is dependent on forming and accessing memories of recent sensorimotor experiences (25–27). Findings from motor learning studies suggest that motor memory may be atypical in autistic individuals, which may contribute to motor control deficits. Autistic individuals show a stronger adaptation to proprioceptive errors than typical controls (4, 14, 15) as well as reduced sensitivity to visual errors during tests of oculomotor adaptation (15, 28, 29). These findings suggest that autistic persons may be more biased toward updating internal models based on proprioceptive feedback than neurotypical controls but may be deficient in using visual feedback or visual-motor memories to update internal models. In a more explicit test of visuomotor memory, autistic participants are less accurate when making saccades to remembered target locations (30–32). These findings suggest that deficits in visuomotor memory, rather than just the integration of visual feedback, may be contributing to sensorimotor impairments in autistic persons. Still, few studies have assessed the role of short-term visuomotor memory during sustained motor control in ASD.

Visuomotor feedback and visuomotor memory processes have been well characterized in studies of precision gripping (27, 27, 33, 34), and precision gripping tasks have repeatedly revealed visuomotor impairments in autistic individuals (10, 17, 22, 23, 35). During visually guided precision gripping, the visuomotor feedback loop lasts approximately one second, and includes integration of visual feedback error information since the last motor command and the execution of a corrective movement based the accumulated visual feedback information (27, 33). In healthy adults, removing visual feedback during precision gripping leads to a decay in force output beginning .5–1.5s after the removal of visual feedback (27). The maintenance of force output for .5–1.5s is consistent with the duration of the visuomotor feedback loop and reflects the capacity for visual feedback error information to be stored in short-term memory. Additionally, force variability and regularity increase in the absence of visual feedback (33, 36, 37), with greater effects over time following the removal of visual feedback (33), indicating that corrective motor commands become less accurate and less dynamic as the motor memory fades.

During a visuomotor memory test in which visual feedback was removed and individuals were instructed to continue gripping at a constant force level, we previously documented a faster rate of force decay among autistic individuals relative to age-, IQ-, and sex-matched neurotypical controls implicating deficient motor memory (35). That study did not analyze age-related effects on motor memory or visuomotor processes, though there are known developmental changes in visuomotor and memory guided motor control throughout childhood and adolescence (36, 38, 39). The development of motor memory and visuomotor feedback processes and their contributions to sustained motor control issues in ASD are still not known.

Here, we aimed to assess differences in short-term visuomotor memory and visual feedback processes during sustained fine motor control in ASD as a function of age. We examined age-associated differences in motor precision, variability, and regularity during visually guided and memory guided precision gripping to test the hypothesis that age-related differences in visuomotor memory and visual feedback processing contribute to sustained fine motor issues in ASD. To further assess motor memory, we examined the latency, slope, and magnitude of force decay following the removal of visual feedback (memory-guided precision gripping). To examine whether motor behaviors vary as a function of the severity of clinical traits, we also examined associations between motor control and clinical ratings of ASD severity, motor behavior, and IQ.

## Methods

### Participants

Fifty-four autistic participants (16 females) and 31 neurotypical (NT) controls (18 females) matched on age (range 10–25 years) completed tests of precision gripping with their dominant hand (Table 1). Autistic participants were recruited through our research registries comprised of individuals evaluated through the University of Kansas Health System who have consented to be contacted for research purposes, and though community advertisements. NT controls were recruited through community advertisements. ASD diagnoses were confirmed based on Diagnostic and Statistical Manual of Mental Disorders, Edition 5 (DSM-5) (40) criteria and classification criteria from the Autism Diagnostic Observation Schedule, Second Edition (ADOS-2) (41) and Autism Diagnostic Interview – Revised (ADI-R) (42). Autistic participants were excluded if they had a known genetic or metabolic disorder associated with ASD (e.g., Fragile X syndrome) or a full scale IQ (FSIQ) below 60 as measured using the Wechsler Abbreviated Scales of Intelligence, Second Edition (WASI-II) (43). NT participants were excluded if they scored ≥ 8 on the Social Communication Questionnaire (44), reported a history of psychiatric or neurologic disorders, had a family history of ASD in first- or second-degree relatives, had a family history of a developmental or learning disorder, psychosis, or obsessive compulsive disorder in first-degree relatives, or had a FSIQ below 85 as measured using the WASI-II. Participants also were excluded if they had a history of head injury with neurological sequelae, birth injury, or seizure disorder. No participants were taking medications known to affect sensorimotor behavior, including antipsychotics, stimulants, or anticonvulsants at the time of testing (45). All participants had corrected or uncorrected visual acuity of at least 20/40. Adult participants provided written informed consent after a complete description of the study, in accordance with the Declaration of Helsinki and the approved Institutional Review Board study protocol (IRB#: STUDY00140269). For participants under the age of 18 and adults who were under legal guardianship, a parent or legal guardian provided written informed consent, and the participant provided written assent. All study procedures were approved by the local Institutional Review Board.

### Clinical Assessments

Participants completed the Wechsler Abbreviated Scales of Intelligence, Second Edition (WASI-II) to assess verbal IQ (VIQ), perceptual IQ (PIQ), and full-scale IQ. The WASI-II is validated for individuals aged 6–89 years. For this study we report scores for the full-scale IQ value that is calculated from all four of the administered subscales, with the exception of two autistic participants who did not complete all four subtests. For these two participants, the two subtest scores are used.

Autistic participants completed the Autism Diagnostic Observation Schedule, Second Edition (ADOS-2) (41) to confirm diagnosis and to quantify severity of autism for analysis. The ADOS-2 is a semi-structured play-based assessment of autistic traits that is the gold-standard diagnostic assessment for autism. It was conducted by a trained research reliable study clinician. Participants in our study were administered module 2 (phrase speech), 3 (verbally fluent children), or 4 (verbally fluent adolescents and adults) according to age and language abilities. The composite severity score (ADOS-CSS) is a standardized score indicating severity of autism that can be compared across modules (higher scores indicate greater severity). The ADOS-CSS are reported and used for analyses.

Autistic participants completed the Repetitive Behavior Scale – Revised (RBS-R) (46, 47), to assess restricted and repetitive behaviors associated with autism. The RBS-R is a questionnaire that asks individuals to rate items from five categories of repetitive behavior (motor stereotypy, self-injurious behavior, compulsions, routines/sameness, and restricted interests). Parents or caregivers completed the RBS-R for participants under 18 years of age, and adult participants completed it as a self-report questionnaire. Higher scores indicate more severe repetitive behavior.

To determine handedness, participants completed the Annett Hand Preference Questionnaire (Annett) (48). The Annett is a 12-item questionnaire that asks the participant which hand they prefer to use for various daily activities (e.g., writing, throwing, using a hammer, etc.), with endorsement of left hand, right hand, or either hand. If left hand is endorsed more than right hand, the participant is considered left-handed, and vice-versa for a classification of right-handed. If left and right hand are equally endorsed, the participant is classified as mixed-handed. Two neurotypical controls scored as mixed-handed and two autistic participants did not complete the Annett, so their dominant hand for precision grip testing was determined based on which hand they used for writing. One control scored as left-handed on the Annett but self-reported as right-handed and used right hand for writing, so they completed precision grip testing with right hand. Handedness counts in Table 1 are based on the hand used to complete precision grip testing.

Participants completed the Bruininks-Oseretsky Test of Motor Proficiency, Second Edition (BOT-2) to assess motor abilities. The BOT-2 is a structured skill-based motor assessment. Participants completed a series of structured motor tasks from three areas: Fine Manual Control, Manual Coordination, and Body Coordination. Composite scores from the Fine Manual Control tests are reported and analyzed for the present study. Higher scores on the BOT-2 reflect better motor performance.

### Precision grip testing

**Figure 1:**
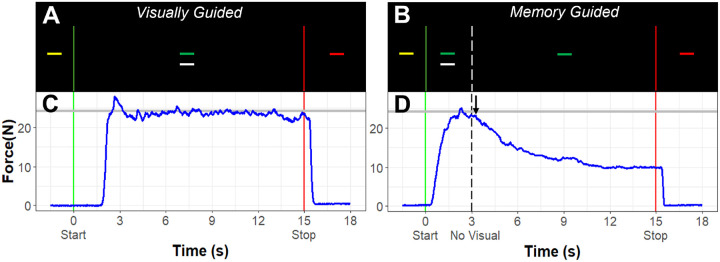
Task Design. **A)** During visually guided trials, participants see a target bar that turns from yellow to green to indicate that they should start pressing. Participants also view feedback of their force output (white bar) for the entire trial. B) During memory guided trials, participants see visual feedback of their force output (white bar) and the green target bar for the first 3s of the trial, after which the white force bar disappears, and they are instructed to keep pressing at the same force level until the target turns red (12s later). C) Example force output (dark blue) for a visually guided trial. The grey line represents target force. D) Example force output (dark blue) for a memory guided trial with target force indicated by the grey line. The participants’ force usually begins to decay (black arrow) after the visual feedback disappears.

Participants completed tests of precision gripping while seated 52cm from a 67cm (27in) Samsung liquid crystal display monitor with a resolution of 1920×1080 and a 120 Hz refresh rate (Fig. 1). Participants sat with the elbow of their dominant hand comfortably positioned at 90° and their forearm resting in a custom arm brace fixed to the table to provide stability during testing. The participants used their thumb and index finger of their dominant hand to press against two opposing precision load cells that were secured to a custom grip device attached to the arm brace. A Coulbourn (V72–25) resistive bridge strain amplifier received analog signals from the load cells, which were converted to digital signals sampled at 100 Hz with a 16-bit analog-to-digital converter (NI USB-6341; National Instruments Corporation). During the first part of the study, ELFF load cells (ELFF-B4–100N; Entran) 1.27cm in diameter were used. Due to normal wear, the ELFF loadcells were replaced with Honeywell loadcells (Model 53, Honeywell International, Inc.) 1.5cm in diameter during the course of the study. ELFF load cells were used for 35.3% of participants (35.5% of controls, 35.2% of autistic participants), and Honeywell load cells were used for 65.5% of participants (64.5% of controls, 64.8% of autistic participants. The voltage-to-Newton calibration was different for each type of loadcell, so a correction calculated from known weights was applied to the force trace after data collection to correct for calibration errors. Additionally, loadcell type was included as a covariate in our analyses to account for differences in loadcell design and calibration effects, including the visual angles of feedback during the task (described below).

Prior to precision grip testing, participants completed an assessment of their maximum grip force, or maximum voluntary contraction (MVC) using their dominant hand. Participants completed three trials in which they were asked to press as hard as they could for three seconds. The average of the participant’s maximum force output across these trials comprised their MVC. For the precision gripping tasks, the target force was set at 45% of the participants’ MVC to account for differences in strength across participants.

During the precision gripping task, participants viewed two horizontal bars on the screen (Fig. 1B). A horizontal white force bar moved upward with increased force and downward with decreased force, and a static bar representing the target force was red during periods of rest. The target bar turned yellow to cue the participant to get ready for the start of the trial, and it turned green to cue the participant to begin pressing at the beginning of each trial. Participants were instructed to press the load cells as quickly as possible when the yellow target bar turned green and to keep pressing so that the white force bar stayed as steady as possible at the level of the green target bar until the target bar turned red, marking the end of the trial.

To test the impact of visual feedback and motor memory processes on grip force behavior, participants completed precision grip testing with and without visual feedback. During visual feedback trials, visual feedback was presented continuously throughout the 15s trial. Due to calibration differences for the two types of loadcells that were used during the study and variance in the distance between the screen and the participants’ eyes during naturalistic viewing, the visual angles ranged from .74 to 1.15 degrees per 1N increase in force output. Visual angles between .623–2.023 degrees result in small changes in the spatial amplitude of visual feedback and small changes in force error (Coombes et al 2010). This range is also associated with stable and optimal variability and regularity of grip force in autistic individuals and NT controls (10).

For the trials without visual feedback (“memory guided” trials), the initial part of the trial was the same as for the visually guided trials – the target bar turned from yellow to green, and the participant pressed on the force transducers to match the white bar to the level of the green target bar. After three seconds, the white force output bar disappeared, and the participant was instructed to continue pressing at the same level until the target bar turned red (12s after the visual feedback was removed). Participants completed blocks of five trials of each condition using their dominant hand (5 trials × 2 conditions = 10 trials). Trials were 15s in duration and alternated with 15s rest periods. Each block was separated by 30s of rest. The target force was set to 45% of the participant’s MVC for all trials. The order of the blocks was pseudorandomized and counterbalanced across participants.

### Data processing

Grip force data were processed using custom applications developed by our lab in R and MATLAB (MathWorks, Inc., Natick, Massachusetts). Trials were excluded if the load cells were not properly re-zeroed between trials or if there were indications that the participant was not following instructions (e.g., the mean force exceeded twice the target force, there was evidence that the participants used fingers other than dominant hand index finger and thumb to press, participants stopped pressing during the trial). For the memory guided condition, trials were excluded if the participant did not reach a stable level of force output within ± 2 Newtons of the target force before visual feedback was removed. Based on these criteria, 19.8% of trials were excluded for the ASD group (12.3% of visually guided and 26.5% of memory guided feedback trials) and 1.3% of trials were excluded for the control group (1.9% of visually guided and .7% of memory guided trials). Trial-level data were averaged for each participant within each condition. Participants needed to have at least two useable trials of a condition for their data to be included. Four autistic participants were excluded from analyses due to insufficient data. An additional five autistic participants had insufficient data for only the memory guided condition, and one had insufficient data for only the visual feedback condition. Final analyses included 50 autistic participants and 31 NT controls with valid data for at least one condition.

To compare force output across conditions, the sustained force output for each trial was analyzed. To account for the differences in trial structure between conditions, only the last 12s of each trial were used for analysis of sustained force output. This 12s phase corresponds to the segment of the memory guided trials where visual feedback was not available and the analogous segment of the trials with visual feedback.

The force traces for each trial were low-pass filtered via a double-pass fourth-order Butterworth filter at a low-pass cutoff of 15 Hz following previous studies from our lab (18, 22). Sustained force data were linearly detrended to account for drift in participants’ force output over the duration of the trial. The mean force of the sustained force data divided by the target force was used as a measure of force accuracy, such that values close to 1 reflect greater accuracy. To assess force variability, the standard deviation (SD) of the force time series was examined. To test the time dependent regularity of the force time series, sample entropy (SampEn) was calculated for each trial (49, 50). SampEn is defined as the natural logarithm of the conditional probability that two similar sequences of *m* data points in a timeseries of a given length *(N)* remain similar within a tolerance level *(r)* at the next data point in the series. SampEn returns a value between 0 and 2. Lower values of SampEn indicate greater regularity of the timeseries (e.g., a sine wave, with its predictable oscillating pattern, would have a SampEn value near 0). Parameter settings for SampEn calculations were *m* = 2 and *r* = .2 × SD of the timeseries. The timeseries length was 1200 data points (12s sampled at 100 Hz). The sampenc.m function (for MATLAB) from the PhysioNet Toolbox (51, 52) was used to calculate SampEn values for each trial.

To characterize the trajectory of force output after visual feedback was removed during the memory guided trials, models were fit to the force traces for each trial. The model consisted of three segments: 1) a horizontal line fit to the stable force output at the beginning of the trial, starting before visual feedback was removed, 2) a logarithmic function fit to the decay in force output after visual feedback was removed, 3) for trials where participants reached a stable force output after their force decayed and before the end of the trial, a horizontal line was fit to the data to model this secondary stable force output. Latency, slope, and magnitude of the force decay were analyzed. Decay latency was measured as the difference between the removal of visual feedback and the beginning of the logarithmic decay model segment. The decay slope was calculated as the log slope of the logarithmic decay model segment. For 2.4% of trials (ASD: 2.5% and NT 2.0%), the force showed little to no decay and was best modeled using a linear fit rather than a logarithmic fit. Descriptive statistics were calculated and reported for the trials with linear decay slopes, but these trials were not factored into the participants’ trial averages or the linear regression models, as they are not directly comparable to the trials with logarithmic slopes. The magnitude of decay was calculated by taking the difference between the force output at the onset of the logarithmic (or linear) decay model segment and the end of the decay model segment or the end of the trial, if force did not stabilize before the end of the trial. This value was then converted to percentage of target force by dividing the raw difference by the participant’s target force to account for the differences in initial force.

### Statistical Analysis

Force accuracy, SD, and SampEn were analyzed using separate linear multilevel mixed effects models (MLM) (53, 54) with the lme4 package in R version 4.0.0 (53). MLM allows for the analysis of within- and between-subjects fixed effects while allowing within-subjects effects to vary randomly and is robust to missing data. Task condition (visually guided, memory guided) was included as a level 1 predictor. Group (ASD, NT) and age were included as level 2 predictors. For all dependent variables, the models also included a two-factor covariate to account for different load cells used during testing (“load cell type”). Random intercepts of participant also were included in the models.

Initial models for force accuracy, SD, and SampEn included the three-way interaction of group × task condition (visually guided, memory guided) × age, all relevant two-way interactions and main effects terms, as well as the covariate for load cell type. To maintain the most parsimonious models possible, other 3- way interactions were not included. Models were fit using the maximum likelihood approach to allow for model comparisons. Terms were removed systematically, and model fit was compared between the previous model and the model with the removed term using likelihood ratio tests. Terms that did not significantly improve model fit (p < 0.05), based on the model comparisons, were not included in the final models. Satterthwaite’s method was used to calculate degrees of freedom for the final model and post hoc comparisons (55). Due to the inherent challenge in determining denominator degrees-of-freedom and calculating p-values for MLMs, we treated the t-value as a z-value and used a z > 1.96 threshold as an additional guideline for determining whether terms explained significant variance in the model (55).

The latency, slope, and magnitude of force decay following the removal of visual feedback were analyzed using separate linear regression models with the lm (linear model) function in R. Group (ASD, NT), age, and the group × age interaction were included as predictors. For all dependent variables, the models also included a covariate for load cell type.

Simple coding was used for group (NT = −0.5, ASD = 0.5), task condition (memory guided = −0.5, visually guided = 0.5), and sex (male = −0.5, female = 0.5). Age was log_10_ transformed. SD, SampEn, and decay magnitude were log_10_ transformed and decay slope and decay latency were square root transformed to correct for skewed distributions. Based on this coding system, the intercept for each model represented the grand mean of the sample.

Pearson correlations were used to assess the relation between experimental variables and ASD symptom severity measured using the ADOS Composite Severity Score (ADOS-CSS) as well as repetitive behaviors measured using the RBS-R. Pearson correlations also were used to assess the relation between visuomotor and motor memory behaviors and IQ for each group.

To determine whether visual feedback guided motor control and motor memory during precision gripping relate to clinically relevant fine motor skills, Pearson correlations were run between the motor variables and BOT-2 Fine Manual Control subscale scores. For each set of correlations, p-values were adjusted using false discovery rate (FDR) to limit Type I error.

## Results

### Force Accuracy

**Figure 2:**
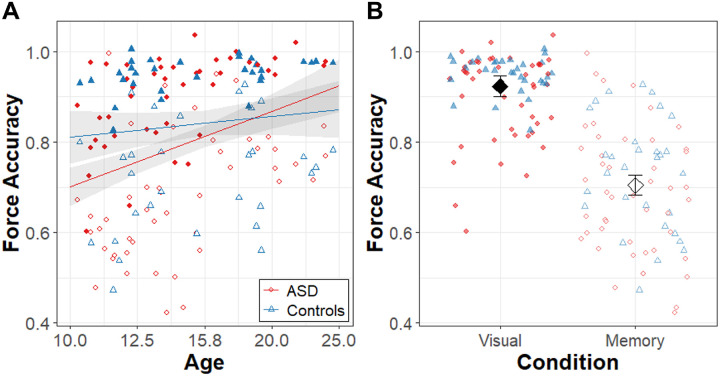
Force accuracy. **A)** Age (log_10_ scale) associations with force accuracy for the ASD (red circles) and NT (blue triangles) groups**. B)** Force accuracy during visually guided (filled points) and memory guided (empty points) precision gripping. Black diamonds represent condition means adjusted for random intercepts of subject in the LMER models. Error bands (A) and bars (B) represent the 95% confidence intervals from the MLM models after accounting for random intercepts of participant.

Figure 2 shows results for force accuracy, and the model summary is reported in Table 2. Group differences in force accuracy varied as a function of age (β = .41, R^2^ = 0.043, t_79.3_ = 2.36, p = 0.021). Follow-up comparisons revealed that autistic individuals showed a greater increase in accuracy with age than the control group (slope_ASD_ = .56 ± .12, slope_NT_ = .15 ± .14). Across ages and groups, force accuracy was greater in the visually guided condition relative to the memory guided condition (β = − .22, R^2^ = 0.563, t_76.7_ = −15.74, p < 0.0001).

### Force Variability

**Figure 3:**
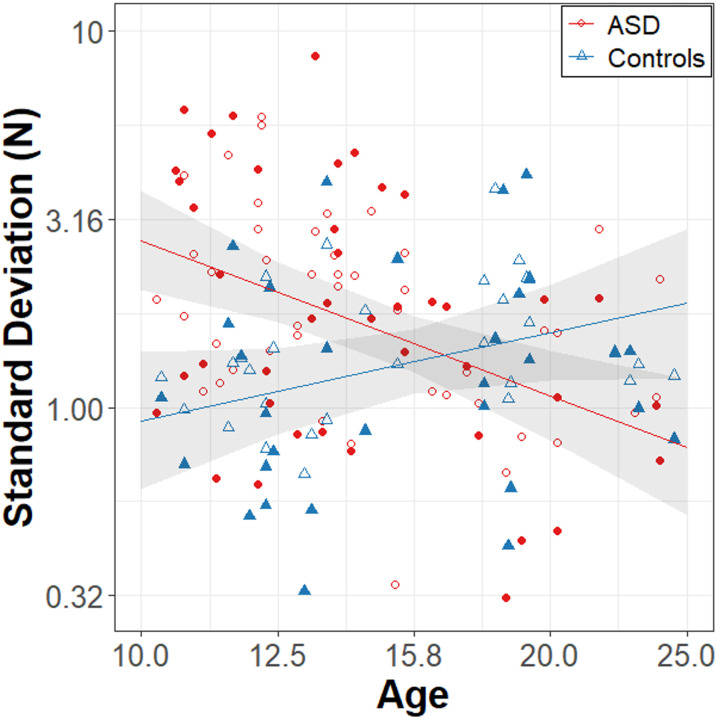
Force Variability in Newtons (N). **A)** Age (log_10_ scale) associations with force standard deviation (log_10_ scale) for the ASD (red circles) and NT controls (blue triangles) groups. Effects did not vary by condition, so data were collapsed across the visually guided (filled points) and memory guided (empty points) conditions. Error bands represent the 95% confidence intervals from the MLM models after accounting for random intercepts of subject.

Results of the model for force SD are summarized in Table 3 and Fig. 3. Group differences in force variability varied as a function of age (β = −2.16, R^2^ = .143, t_77.1_ = −4.04, p = 0.0001). Follow-up comparisons revealed that the ASD group showed a stronger age-related decrease in force SD than the control group (slope_ASD_ = −1.38 ± .35, slope_NT_ = .78 ± .43). Overall, the ASD group showed higher force SD than the control group (β = 2.64, R^2^ = .151, t_77.2_ = 4.18, p < 0.0001). No effects of task condition were observed.

### Force Regularity

**Figure 4:**
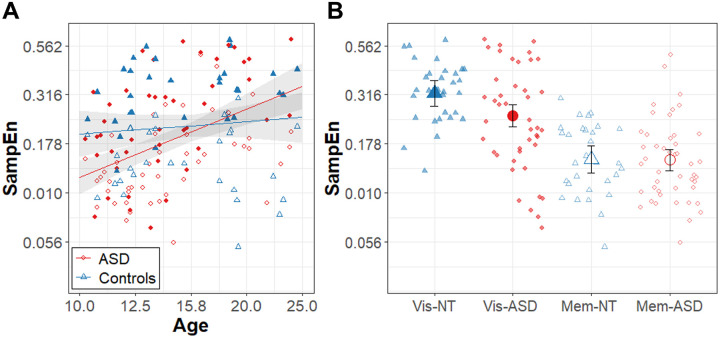
Force Regularity. **A)** Age (log_10_ scale) associations with force SampEn (log_10_ scale) for the ASD (red circles) and NT controls (blue triangles) groups. Higher SampEn corresponds to lower regularity**. B)** Force SampEn for the ASD (red circles) and NT controls (blue triangles) groups during the visually guided feedback (Vis; solid points) and memory guided (Mem; open points) conditions. Error bands and bars represent the 95% confidence intervals from the MLM models after accounting for random intercepts of subject.

Results of the model for force SampEn are summarized in Table 4 and Fig. 4. Group differences in force SampEn varied as a function of task condition (β = .11, R^2^ = .021, t_75.0_ = 2.24, p = 0.028) and age (β = .95, R^2^ = .064, t_79.6_ = 2.75, p = 0.007). Follow-up comparisons revealed that the ASD group showed a greater age-related increase in force SampEn than the control group (slope_ASD_ = 1.16 ± .23, slope_NT_ = .21 ± .29), and the ASD group showed lower SampEn than controls only in the visually guided condition (mean_ASD_ = −0.61 ± .03, mean_TD_ = −0.50 ± .04). Across groups, the effects of condition on force SampEn varied as a function of age (β = − .52, R^2^ = .021, t_77.4_ = −2.21, p = 0.030). Follow-up comparisons revealed that age related increases in SampEn are stronger in the visually guided condition relative to the memory guided condition (slope_Visual_ = .95 ± .22, slope_Memory_ = .43 ± .22).

### Slope of Force Decay

**Figure 5:**
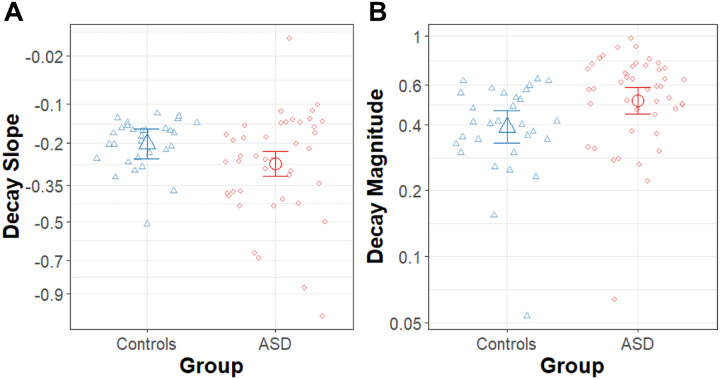
Decay Slope and Magnitude. **A)** Slope of the force decay (square root scale) following the removal of visual feedback for the ASD (red circles) and NT controls (blue triangles) groups. B) Magnitude of the force decay (log_10_ scale) following the removal of visual feedback for ASD (red circles) and NT controls (blue triangles). Large points represent group means adjusted for random intercepts of subject in the MLM models. Error bars represent the 95% confidence intervals from the MLM models after accounting for random intercepts of subject.

The results of the linear model for the slope of force decay in the memory guided condition are summarized in Table 5 and Fig. 5. In the model for the slope of force decay the ASD group had steeper (more negative) decay slope than the NT group (β = − .07, R^2^ = .001, t_71_ = −2.06, p = .044) (mean_ASD_ = −0.51 ± 02, mean_NT_ = −0.45 ± 03). In the ASD group, seven of 275 (2.5%) trials were fit with a linear slope (mean = − .00006 ± .00011), and in the control group, three of 150 (2.0%) trials were fit with a linear slope (mean = − .00023 ± .00025), indicating that each group had a comparable proportion of trials that showed little to no decay.

### Decay Latency

In the model for the latency of force decay onset, no terms were significant. For reference, Table 6 shows the model summary for the model containing nonsignificant main effects of Group and Age, as well as the covariate for the different load cells used over the course of the study. To interpret findings of the decay onset latency in the context of short-term visuomotor memory processes, we calculated the group medians of the raw (untransformed) latency values. Median decay onset latencies were .842s for ASD and .669s for NT. Median values are reported due to a rightward skew in the distribution of the untransformed latency data (skewness = 1.35).

### Decay Magnitude

Results of the model for the magnitude of the force decay in the memory guided condition are summarized in Table 7 and Fig. 5. The magnitude of decay was calculated as a proportion of the participant’s target force with larger values representing greater decay. The values were log_10_ transformed to correct for skewed distributions. The ASD group had a greater magnitude of force decay than the NT group (β = − .12, R^2^ = .08, t_71_ = 2.53, p = .014) (mean_ASD_ = −0.29 ± .03, mean_NT_ = −0.41 ± .04). Additionally, the magnitude of force decay decreased significantly with age (β = −0.86, R^2^ = .154, t_71_ = −3.59, p = 0.0006)

### Relation to Clinical Features

#### Autism severity:

**Figure 6:**
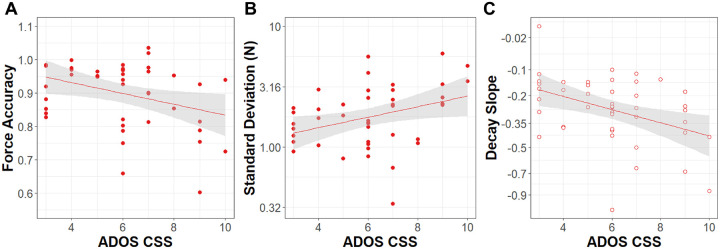
Relation of ASD Symptomatology to grip control. Association between ADOS-CSS scores and **A)** force accuracy and **B)** force variability in Newtons (N; Standard deviation (log_10_ scale)) during visually guided precision gripping. **C)** Association between ADOS-CSS scores and the slope of logarithmic force decay during memory guided precision gripping. Error bands represent standard error.

More severe clinical ratings of autism on the ADOS-CSS were positively correlated with SD (r = .49, p_FDR_ = .009) and negatively correlated with force accuracy (r = − .34, p_FDR_ = 023) in the visually guided condition (Fig. 6). ADOS-CSS was negatively correlated with decay slope in the memory guided condition (r = − .41, p_FDR_ = .029). No other dependent variables correlated with ADOS-CSS for either condition. Scores on the RBS-R were not significantly correlated with any measure of grip force control.

#### IQ:

FSIQ, VIQ, and PIQ were not significantly correlated with any of the dependent variables for either group or feedback condition.

#### Fine Motor Behavior:

For the ASD group only, BOT Fine Manual Control scores were negatively correlated with force SD in the visually guided condition (r = − .50, p_FDR_ = .014). The ASD group also showed trending correlations of BOT Fine Manual Control scores with force accuracy (r = .37, p_FDR_ = .095) in the visually guided condition after FDR corrections. No other dependent variables were significantly correlated with BOT Fine Manual Control scores in either group or condition.

## Discussion

The present study assessed the unique contributions and age-dependent patterns of visual feedback processing and motor memory to well-established differences in sustained precision motor control in autistic individuals. We replicated our prior finding (35) that autistic individuals show a faster rate of decay in their grip force than NT controls following the removal of visual feedback, suggesting deficient motor memory contributes to sensorimotor impairments in autistic individuals. Additionally, we observed greater age-associated improvements in sustained motor control in autistic individuals relative to NT individuals reflecting reduced abilities during early childhood followed by normalization of sensorimotor control during early adolescence and early adulthood. These results suggest sensory feedback processes that contribute to sensorimotor precision follow a protracted course of maturation in ASD. Age-related improvements were not specific to visually guided or memory guided motor control, suggesting that developmental delays may impact multiple motor control mechanisms in ASD.

### Motor memory differences in ASD

During memory-guided grip control, force decayed at a greater rate and to a greater extent in autistic individuals compared to NT controls. These findings are consistent with our prior study (35). However, we did not find group differences in the latency of force decay after the removal of visual feedback. The visuomotor memory only lasts .5–1.5s following the removal of feedback, so latencies that exceed .5–1.5s would reflect the involvement of motor memory processes for maintaining force output that are distinct from short-term visuomotor memory processes (27, 33). For both groups, the decay onset latencies were well within the range of visuomotor memory (median_ASD_ =. 842s; median_NT_ = .669s), supporting the interpretation that short-term visuomotor memory is not impaired in ASD in the context of visuomotor control. Together, these findings indicate that motor memory processes are impacted in ASD, but they are independent of short-term visuomotor memory processes. Greater and more rapid decay of the motor memory in autistic individuals may result from reduced reliance on somatosensory feedback during visually guided precision gripping relative to NT controls (22). In the absence of visual feedback, somatosensory feedback is critical for monitoring motor output during precision gripping. Reduced integration of somatosensory feedback during the formation of the motor memory (when visual feedback is available) may limit autistic individuals’ ability to maintain force output at a consistent level after visual feedback is removed and somatosensory feedback becomes the primary sensory input.

### Age-related differences in visuomotor control among autistic individuals

Autistic individuals showed greater regularity of grip force than controls, specifically during visually guided precision gripping, consistent with previous studies of visually guided precision gripping in ASD (10, 22). Regularity represents the degrees of freedom of movement. Lower regularity (higher entropy) indicates greater processing and integration of sensory feedback information for updating ongoing motor behaviors. This finding is consistent with prior studies (10, 22) and suggests that autistic individuals do not integrate visual feedback as effectively as controls to optimize control of precision movements (22). Force regularity was the only variable for which condition effects varied as a function of group. This could indicate that force regularity is more sensitive to group differences in visual feedback and motor memory processes than motor variability or accuracy, as regularity has been shown to be more sensitive to slight changes in visual feedback during visually guided gripping than variability (33). Alternatively, these findings could indicate that autistic individuals and controls achieve comparable high accuracy and variability through different mechanisms, though future studies are needed to test these hypotheses.

Autistic individuals also show stronger age associations than the NT group for all sustained force variables. In the ASD group, force variability and regularity decreased with age and force accuracy increased with age, while these metrics were stable across ages in the NT group. Specifically, younger autistic children showed poorer force control relative to NT controls, while adolescents and young adults were comparable across groups. These findings are consistent with our prior study of sustained visually guided precision gripping showing that sensorimotor impairments are more robust in ASD at younger ages (22). However, in a separate study that included a broader age range (5–35 years), we found stronger age-related improvements in grip force regularity in controls relative to autistic individuals and comparable age-associations in force variability across groups at the visual angle and target force level used in the present study. These findings are likely driven by the rapid maturation of motor processes in neurotypical development that occur at younger ages than were included in the present study, as well as variation across autistic individuals in the extent to which sensorimotor processes are disrupted. Together, these findings suggest that autistic individuals have delayed development of sustained, precision motor control processes relative to neurotypical individuals. These age associations did not differ across task conditions, indicating that delayed motor development impacts multiple motor processes and is not specific to visuomotor control.

While literature tracking developmental trajectories of motor function in ASD from childhood to adulthood is sparse, some studies suggest a normalization or improvement in motor skills with age in autistic individuals. Young autistic children showed elevated variability in stride velocity, stride time, and stride length relative to relative to neurotypical children during gait, but these differences were not present during adolescence (56). Autistic children show deficits in reach-to-grasp behaviors, including larger normalized jerk score and more motor units than NT children (57), but a separate study using similar methods did not find differences between autistic and neurotypical adults (58). While these studies are consistent with our findings of age-related normalization of motor skills in autistic individuals, longitudinal studies are needed to understand the developmental trajectories of discrete sensorimotor processes in autism.

### Neurophysiology of visuomotor and motor memory processes

Precision visuomotor behaviors rely on cerebellar-cortical brain networks. Visual and somatosensory inputs are integrated in posterior parietal cortex (superior and inferior parietal lobules) (59–61) and are relayed to premotor and primary motor cortices to generate motor commands (62–64). Lateral (Crus I-II), anterior (I-V), and posterior (VIIb) cerebellar lobules receive efferent copies of the motor commands from primary motor cortex, containing information on the expected sensory consequences of the movement, and compare them to the actual sensory consequences of the behavior which are received from primary and association sensory cortices (65, 66). Based on the discrepancy between the actual and expected sensory consequences of the movement, the cerebellum issues a corrective motor command, which is relayed to the primary motor cortex through the thalamus (34, 67). More diffuse cortical-subcortical networks including basal ganglia (putamen), supplemental motor area, and anterior prefrontal cortex have also been shown to be involved in visuomotor transformations (34).

Motor memory processes rely on distinct, but overlapping brain networks which include dorsolateral and ventral prefrontal cortices, ventral premotor cortex, and anterior cingulate cortex (error monitoring) (34, 68). Immediately following the transition from visually guided to memory guided grip control, contralateral ventral premotor cortex (300–500ms) and (anterior) ventral prefrontal cortex (400ms) show responses (event-related potentials) that do not occur in conditions where the resolution of visual feedback changes dramatically but is still present, indicating that these responses are specific to motor memory processes (68). Visual and contralateral motor cortices also show changes in activation immediately following the removal of visual feedback (300–400ms). These changes in brain activation are evident prior to changes in behavior, which occur at around 600ms, suggesting their likely involvement in short-term visuomotor memory processes. Beyond the temporal capacity of short-term visuomotor memory, activity associated with motor memory processes is isolated to dorsolateral prefrontal, ventral prefrontal, and anterior cingulate cortices contralateral to the hand used for gripping, and activation of these regions during memory guided movements is more medial than during visually guided movements (34).

Our present findings that autistic individuals show deficits in visually guided and memory guided precision gripping are consistent with neuroimaging studies showing atypical activation and connectivity in cerebellar-posterior parietal cortical circuits that translate sensory information into reactive motor adjustments, as well as frontal-cerebellar networks involved in cognitive processing (21, 69). Our functional MRI study of precision gripping revealed increased activation of cortical sensory and motor control regions (e.g., supplementary motor area, superior parietal lobule, middle frontal gyrus, inferior frontal gyrus) in ASD that was more pronounced when visual feedback was amplified as well as reduced parietal-premotor and parietal-putamen functional connectivity, indicating reduced modulation of circuits involved in sensory processing and precision motor control as well as reduced integration of sensory feedback with motor control systems in autistic individuals (17). Further, that neuroimaging study also found that autistic individuals had stronger age-associated increases in functional connectivity of cerebellar-cortical networks involved in sensory processing (visual cortex) and motor control (primary motor and premotor cortices) that were associated with reduced force variability, consistent with the present finding of age-associated increases in precision motor control in ASD.

Neuroimaging studies of ASD have found atypical activation and functional connectivity of brain systems involved in motor memory. At rest, autistic individuals show reduced functional connectivity between cerebellum and prefrontal cortex (21, 69). Additionally, we found reduced functional connectivity between primary motor cortex and anterior cingulate cortex in ASD during visuomotor behavior, specifically under more challenging conditions (70). While these studies demonstrate that autistic individuals show atypical function of brain circuits that have been implicated in motor memory, future neuroimaging studies during memory guided motor behavior in ASD are needed to determine whether behavioral metrics of motor memory impairments in ASD are associated with atypical activation or connectivity within these brain regions.

### Clinical Associations

Clinically rated ASD severity was associated with force regularity and accuracy during visually guided precision gripping and rate of force decay during memory guided precision gripping suggesting that differences in visuomotor integration and motor memory may both contribute to the development of autism. These clinical associations were not observed with the RBS-R or IQ, indicating they may be more relevant to social-communication differences in ASD. Sensorimotor integration is fundamental to the development of social and communication behaviors (71, 72). Early development of sensorimotor processes including the integration of sensory feedback and updating internal models based on the sensory consequences of the individual’s movements are necessary for mapping others’ behavior (e.g., social gestures, facial expressions, speech) onto one’s own sensorimotor representations (71, 72). This process provides information on the timing and intent of the other person’s movements and facilitates the learning of social-communication behaviors through imitation, interpreting others’ social-communication behaviors, and understanding the dynamics of reciprocal social interaction. Given that sensorimotor deficits are some of the earliest indicators of atypical development in ASD, it is possible that they contribute to later deficits in social interaction and communication (73–75).

### Limitations

Very few studies have characterized age-related patterns of sensorimotor behavior and processing across broad ranges in autism. The present study used a cross-sectional design to assess age-associated differences in visuomotor feedback and motor memory processes during sustained precision gripping in autism. However, to better understand developmental trajectories of sensorimotor function in autism, longitudinal studies of multiple distinct sensorimotor processes and effector systems are needed. Additionally, while we observed differences in memory guided motor control in autistic individuals relative to NT controls, we were not able to determine how other sensorimotor control processes – including reliance on somatosensory feedback, attention, or motor learning – may be impacting behavior in this condition. Our prior findings suggested that autistic individuals have reduced reliance on somatosensory feedback for sustained precision gripping than NT controls, which may contribute to the greater and more rapid decay of force we observed during memory guided control in autistic individuals in the present study. However, future studies are needed to identify whether reduced somatosensory feedback processes or other sensorimotor control processes (e.g., learning, attention) may be contributing to differences in memory guided sustained precision motor control in ASD.

## Conclusion

The present study demonstrates that differences in visual feedback and motor memory processing contribute to sustained precision motor control deficits in autistic individuals, though short-term visuomotor memory is unaffected. Precision motor control deficits are most pronounced in childhood and normalize in adolescence and early adulthood suggesting that autistic individuals have protracted development of precision motor control. Our findings provide novel insights into neurodevelopmental processes underlying precision sensorimotor behavior in ASD.

## Figures and Tables

**Table 1:** Demographic and clinical characteristics of autistic individuals (ASD) and neurotypical controls (NT)

		ASD		NT			
	N	Ratio		N	Ratio		OR
**Sex** ^1^	54	38M:16F	-	31	13M:18F	-	.309*
**Handedness** ^2^	54	6L:48R	-	31	4L:27R	-	.845*
	N	Mean	SD	N	Mean	SD	t
**Age**	54	14.87	3.68	31	15.90	4.11	−1.16
**ADOS-CSS**	54	6.26	2.13	-	-	-	-
**RBS-R**	52	32.15	21.75	-	-	-	-
**FSIQ** ^3^	54	97.39	16.47	31	110.51	10.27	−4.52*
**VIQ** ^3^	52	95.40	17.82	31	108.45	10.63	−4.18*
**PIQ** ^3^	53	99.32	17.09	31	110.45	12.47	−3.43*
**BOT-2: Fine Manual Control**	48	41.31	9.35	23	48.43	10.21	−2.83*
**MVC**	54	40.17	17.41	31	49.59	17.65	−2.38*

ASD: Autism spectrum disorder; NT: Neurotypical; OR: Odds ratio from Fisher’s Exact test; M: Male; F: Female; L: Left-handed; R: Right-handed; SD: Standard deviation; ADOS-CSS: Autism Diagnostic Observation Scale – Composite Severity Score; RBS-R: Repetitive Behavior Scale – Revised; FSIQ: Full-scale intelligence quotient; VIQ: Verbal intelligence quotient; PIQ: Perceptual intelligence quotient; BOT-2: Bruininks-Oseretsky Test of Motor Proficiency, 2nd Edition; MVC: Maximum voluntary contraction.

1 Biological sex is used here. Three autistic participants did not identify as the sex they were assigned at birth (all assigned female). One identified as a transgender male; one as non-binary, and one as gender fluid.

2 Handedness here refers to which hand the participant used for precision grip testing. Five participants either did not complete the Annett (two autistic participants) or had scores on the Annett that did not match the hand they used for writing (three controls).

3 Two autistic participants did not complete all four subscales of the WASI-II, so the two-subscale FSIQ was used for those participants, and PIQ and VIQ subscales were omitted for participants who did not complete both subscales required for those scores.

**Table 2:** Linear mixed effects model summary for force accuracy

	Fixed Effects	Estimate (SE)	df	t	Partial R^2^
**Accuracy**	*Intercept*	.40 (.11)	78.4	**3.63** ***	
	*Level 1*				
	Condition	−.22 (.01)	76.7	−15.74***	.563
	Load Cells	− .02 (.02)	77.9	−1.06	.009
	*Level 2*				
	Group	−0.52 (.20)	79.4	−2.53*	.050
	Age (log10)	.36 (.09)	78.4	**3.82** **	.106
	*Interactions*				
	Group × Age (log_10_)	.41 (.17)	79.3	**2.36** *	.043
	Random Effects	Variance (SD)			
	*Participant (intercept)*	.002 (.047)			
	Residual	.007 (.086)			

SD: Standard Deviation; SE: standard error.

* p < .05,

** p < .01,

*** p < .001

**Table 3:** Linear mixed effects model summary for force variability (SD)

	Fixed Effects	Estimate (SE)	df	t	Partial R^2^
**SD (log**_**10**_)	*Intercept*	.49 (.34)	76.3	1.42	
	*Level 1*				
	Condition	.04 (.03)	71.1	1.45	.006
	Load Cells	.11 (.06)	76.8	1.85	.034
	*Level 2*				
	Group	2.64 (.63)	77.2	**4.18** ***	.151
	Age (log_10_)	−0.30 (.29)	76.3	−1.03	.011
	*Interactions*				
	Group × Age (log_10_)	−2.16 (.53)	77.1	−4.04***	.143
	Random Effects	Variance (SD)			
	*Participant (intercept)*	.04 (.20)			
	Residual	.03 (.18)			

SD: standard deviation; SE: standard error.

* p < .05,

** p < .01,

*** p < .001

**Table 4:** Linear mixed effects model summary for force regularity (SampEn)

	Fixed Effects	Estimate (SE)	df	t	Partial R^2^
**SampEn (log**_**10**_)	*Intercept*	−1.50 (.22)	78.6	−6.73***	
	*Level 1*				
	Condition	.33 (.28)	77.6	1.18	.006
	Load Cells	.02 (.04)	80.0	.55	.003
	*Level 2*				
	Group	−1.17 (.41)	79.6	−2.87**	.069
	Age (log10)	.69 (.19)	78.7	**3.68** ***	.110
	*Interactions*				
	Group × Condition	.11 (.05)	75.0	**2.24** *	.021
	Group × Age (log_10_)	.95 (.35)	79.6	**2.75** **	.064
	Condition × Age (log_10_)	−0.52 (.23)	77.4	−2.212*	.021
	Random Effects	Variance (SD)			
	*Participant (intercept)*	.01 (.11)			
	Residual	.02 (.15)			

SampEn: Sample Entropy; SD: Standard Deviation; SE: standard error.

* p < .05,

** p < .01,

*** p < .001

**Table 5:** Linear model summary for decay slope

	Fixed Effects	Estimate (SE)	t	Partial R^2^
**Slope (sqrt)**	*Intercept*	−0.68 (.20)	−3.43***	
	*Level 1*				
	Load Cells	−0.04 (.04)	−1.26	<.001
	*Level 2*				
	Group	−0.07 (.03)	−2.06*	.001
	Age (log10)	.17 (.17)	1.02	<.001
	Random Effects	SE	df	
	Residual	.14	71	

Sqrt: square root transform; SD: Standard Deviation; SE: standard error.

* p < .05,

** p < .01,

*** p < .001

**Table 6:** Linear model summary for decay latency

	Fixed Effects	Estimate (SE)	t	Partial R^2^
**Latency (sqrt)**	*Intercept*	1.12 (.53)	**2.10** *	
	*Level 1*			
	Load Cells	−0.03 (.10)	− .31	.001
	*Level 2*			
	Group	−0.05 (.09)	− .53	.004
	Age (log10)	−.03 (.10)	− .31	.001
	Random Effects	SE	df	
	Residual	.38	73	

Sqrt: square root transform; SD: Standard Deviation; SE: standard error.

* p < .05,

** p < .01,

*** p < .001

**Table 7:** Linear model summary for decay magnitude

	Fixed Effects	Estimate (SE)		t	Partial R^2^
**Magnitude (log**_**10**_)	*Intercept*	.66 (.28)		**2.32** **	
	*Level 1*				
	Load Cells	− .03 (.05)		− .54	.004
	*Level 2*				
	Group	.12 (.05)		**2.53** *	.083
	Age (log10)	−0.86 (.24)		−3.59***	.154
	Random Effects	SE	df		
	Residual	.20	71		

SD: Standard Deviation; SE: standard error.

* p < .05,

** p < .01,

*** p < .001

## Data Availability

Data is provided within the manuscript or supplementary information files. Raw data will be made available to researchers upon reasonable request to the corresponding author(s).

## Abbreviations

ASD: Autism spectrum disorder
NT: Neurotypical
IQ: Intelligence quotient
ADOS-2: Autism Diagnostic Observation Schedule, Second Edition
ADI-R: Autism Diagnostic Interview – Revised
FSIQ: Full-Scale IQ
WASI-II: Wechsler Abbreviated Scales of Intelligence – Second Edition
OR: Odds ratio from Fisher’s Exact test
M: Male
F: Female
L: Left-handed
R: Right-handed
SD: Standard deviation
ADOS-CSS: Autism Diagnostic Observation Scale – Composite Severity Score
RBS-R: Repetitive Behavior Scale – Revised
VIQ: Verbal intelligence quotient
PIQ: Perceptual intelligence quotient
BOT-2: Bruininks-Oseretsky Test of Motor Proficiency, 2nd Edition
MVC: Maximum voluntary contraction
SampEn: Sample entropy
MLM: Muli-level mixed effects model
FDR: False discovery rate
SE: Standard error
Sqrt: Square root
